# Next-generation sequencing testing in children with epilepsy reveals novel clinical, diagnostic and therapeutic implications

**DOI:** 10.3389/fgene.2023.1300952

**Published:** 2024-01-05

**Authors:** Magdalena Krygier, Marta Pietruszka, Marta Zawadzka, Agnieszka Sawicka, Anna Lemska, Monika Limanówka, Jan Żurek, Weronika Talaśka-Liczbik, Maria Mazurkiewicz-Bełdzińska

**Affiliations:** Department of Developmental Neurology, Medical University of Gdansk, Gdansk, Poland

**Keywords:** epilepsy, genetics, next-generation sequencing, monogenic epilepsy, developmental and epileptic encephalopathy, neurodevelopmental disorder

## Abstract

**Introduction:** Epilepsy is one of the commonest diseases in children, characterized by extensive phenotypic and genetic heterogeneity. This study was conducted to determine the diagnostic utility and to identify novel clinical and therapeutic implications of genetic testing in pediatric patients with epilepsy.

**Methods:** Large multigene panel and/or exome sequencing was performed in 127 unrelated Polish and Ukrainian patients with suspected monogenic epilepsy. Diagnostic yields were presented for five phenotypic subgroups, distinguished by seizure type, electroencephalographic abnormalities, anti-seizure treatment response, and neurodevelopmental deficits.

**Results:** A definite molecular diagnosis was established in 46 out of 127 cases (36%). Alterations in six genes were detected in more than one patient: *SCN1A*, *MECP2*, *KCNT1*, *KCNA2*, *PCDH19*, *SLC6A1*, *STXBP1*, and *TPP1*, accounting for 48% of positive cases. 4/46 cases (8.7%) were mosaic for the variant. Although the highest rates of positive diagnoses were identified in children with developmental delay and generalized seizures (17/41, 41%) and in developmental end epileptic encephalopathies (16/40, 40%), a monogenic etiology was also frequently detected in patients with solely focal seizures (10/28, 36%). Molecular diagnosis directly influenced anti-seizure management in 15/46 cases.

**Conclusion:** This study demonstrates the high diagnostic and therapeutic utility of large panel testing in childhood epilepsies irrespective of seizure types. Copy number variations and somatic mosaic variants are important disease-causing factors, pointing the need for comprehensive genetic testing in all unexplained cases. Pleiotropy is a common phenomenon contributing to the growing phenotypic complexity of single-gene epilepsies.

## 1 Introduction

Epilepsy is one of the commonest diseases in young children, with estimated incidence exceeding 60 per 100,000 under the age of 5 years (Hauser et al., 1993). It is defined by the enduring predisposition of the brain to generate epileptic seizures with its neurobiologic, cognitive, and psychosocial consequences (Fisher et al., 2005). The etiologies of epilepsies are highly variable and involve structural, genetic, metabolic, infectious, autoimmune, and neurodegenerative causes (Balestrini et al., 2021). Early age of seizure onset is associated with high rates of pharmacoresistancy, as well as behavioral, motor and cognitive comorbidities, which poses a significant socioeconomic burden (Wirrell et al., 2012; Jennum et al., 2017). International League Against Epilepsy (ILAE) defined specific epilepsy syndromes, basing on the presence of characteristic, relatively uniform clusters of clinical and electroencephalographic features, supported by specific etiological findings. Syndromes at various ages were broadly divided into generalized, focal, and combined generalized and focal epilepsies (Riney et al., 2022). In patients with neonatal and infantile disease-onset two major epilepsy syndromes were distinquished: self-limited epilepsies, where there is a high probability of spontaneous remission and developmental and epileptic encephalopathies (DEEs), characterized by developmental impairment caused by both epileptic activity and developmental mechanisms (Zuberi et al., 2022). Determining the cause of epilepsy frequently carries prognostic and therapeutic implications. Describing novel etiology-defined epilepsy syndromes and periodical updates of classifications are essential for proper diagnosis and prompt initiation of syndrome-specific treatment.

It is well established that a substantial proportion of childhood-onset epilepsies have a genetic background. The introduction of next-generation sequencing (NGS) techniques to clinical and experimental epileptology significantly changed our understanding of the complex landscape of epilepsy genetics. Genetic epilepsies represent a large, clinically and genetically heterogeneous group of diseases, arising from monogenic, polygenic or copy number variations (CNVs). It is estimated that up to 40% of patients with severe epilepsy have a single-gene etiology (Guerrini et al., 2021). So far, variations in hundreds of genes have been associated with epilepsy and variable neurodevelopmental deficits. The application of NGS-based tests, such as multigene panels (MGP) and exome sequencing (ES), as well as chromosomal microarray (CMA) in clinical practice allow for a rapid detection of causative variants in a substantial number of patients, especially those with early-onset, drug-resistant seizures and other comorbidities. The highest rate of diagnoses is achieved by genome sequencing, followed by ES but large MGPs are also widely used in clinical setting, enabling a rapid and cost-effective detection of pathogenic variants in around 19%–24% of cases (Stefanski et al., 2021; Sheidley et al., 2022).

Advances in molecular genetic testing revealed not only the wide genetic heterogeneity of certain epilepsy syndromes but also phenotypic pleiotropy, meaning that alterations in the same gene can result in various epileptic and neurodevelopmental phenotypes (McTague et al., 2016). This points to the need for further evaluation of clinical and genetic associations in certain disease entities. In addition, identification and characterization of molecular background of epilepsies across different populations and age groups is essential for developing further diagnostic and therapeutic approaches.

This study presents the results of next-generation sequencing (MGP and/or ES) in 127 consecutive pediatric patients with suspected monogenic epilepsy, referred to a single tertiary epilepsy centre in Poland. Together with detailed clinical and neuroimaging data, these results show novel clinical, molecular and therapeutic implications.

## 2 Material and methods

### 2.1 Cohort recruitement

Patients were recruited in a single tertiary epilepsy centre in Poland (Department of Developmental Neurology, Medical University of Gdansk) from January 2018 to March 2023. In total, 127 unrelated children were enrolled, 121 of Polish and six of Ukrainian origin. Inclusion criteria were any of: (i) child presenting with epilepsy in first year of life, (ii) child with epilepsy and developmental delay, intellectual disability and/or autism spectrum disorder; (iii) child presenting with atypical febrile seizures of febrile status epilepticus; (iv) drug resistant epilepsy; (v) child with positive family history of epilepsy concominant with monogenic inheritance. Patients with other etiologies, that fully explained the presence of seizures, such as autoimmune epilepsies with positive specific antibodies, children with perinatal complications with clinical and radiological signs corresponding to hypoxic-ischemic injury or children with pathogenic CNVs detected by chromosomal microarray were not enrolled to the study. In addition, patients were excluded if clinical and electroencephalographic manifestations were typical for polygenic epilepsies and there were no other neurodevelopmental concerns.

Diagnosis of epilepsy and drug resistant epilepsy was established according to International League Against Epilepsy (ILAE) definitions (Kwan et al., 2010; Fisher et al., 2014). Developmental delay was defined as performance that is two or more standard deviations below the mean on age-appropriate standardized tests in children under 5 years of age in one or more developmental domains: gross or fine motor, speech/language, cognition, social or emotional and adaptative behavior (Bélanger and Caron, 2018). Intellectual disability and autism spectrum disorder were diagnosed according to DSM-5 criteria in specialized multidisciplinary ambulatory services (American Psychiatric Association, 2013).

All patients underwent clinical assessment by the team of paediatric neurologist, clinical geneticist, metabolic paediatrician, and epileptologist. Repeated video-electroencephalography recordings, magnetic resonance imaging, and basic metabolic tests (routine blood laboratory tests, urinary organic acids, plasma amino acids, and acylcarnitines) were performed in all patients. Additional neuroimaging, metabolic and electrophysiological tests were carried out depending on clinical symptoms. Pre- and post-test genetic counseling was performed for each family.

### 2.2 Editorial policies and ethical considerations

This research study adheres to the principles set out in the Declaration of Helsinki. Informed consent for molecular genetic testing and for anonymous data sharing was obtained from each participant and/or legal guardian and the study was approved by the Medical University of Gdansk institutional review board (ID NKBBN/13/2023). In line with the American College of Medical Genetics and Genomics (ACMG) recommendations, the informed consent included the option to receive the report of clinically significant incidental findings when ES was performed (ABo, 2019).

### 2.3 Variant identification and analysis

**FIGURE 1:**
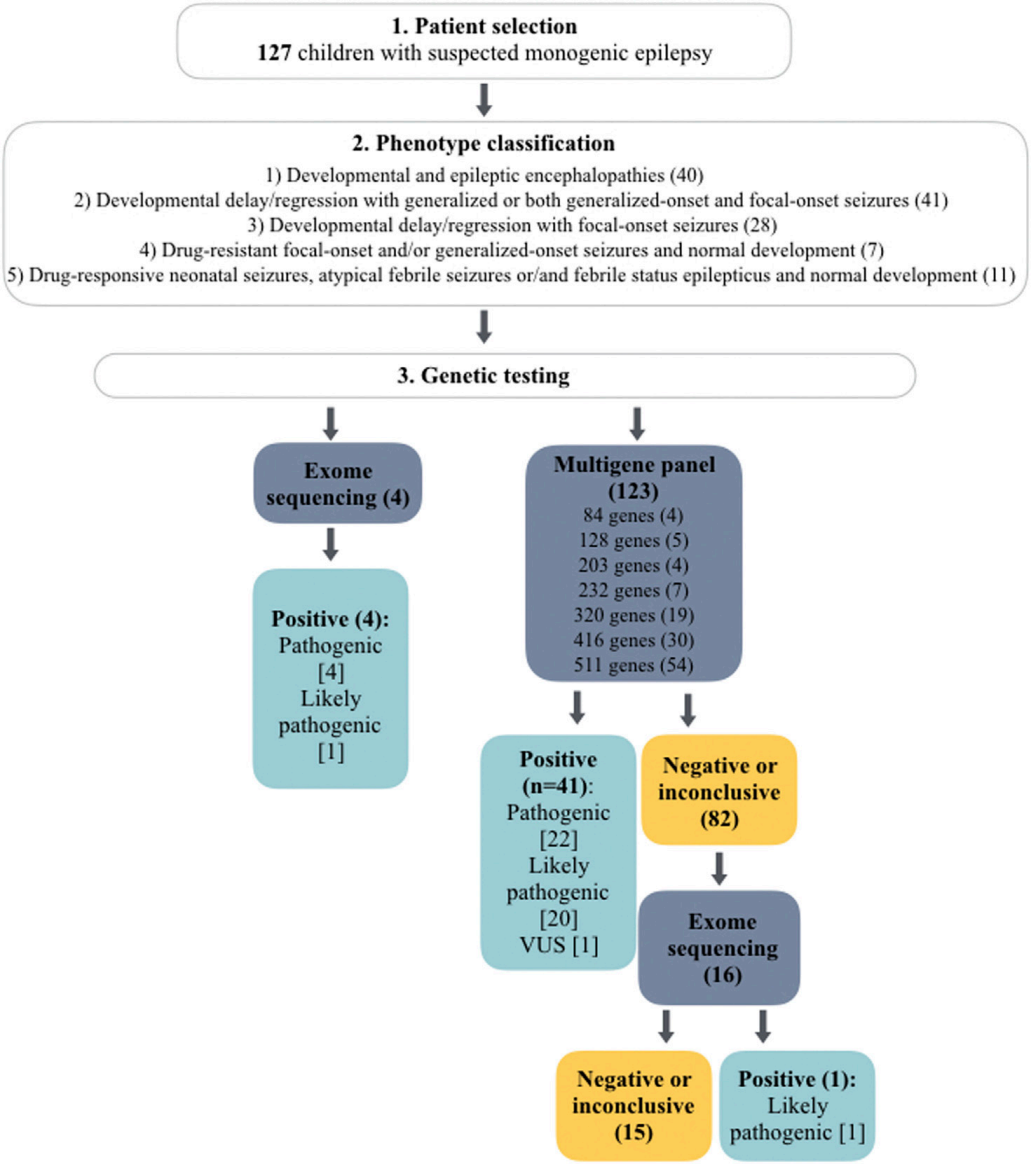
Flowchart of genetic testing outcomes in a total of 127 cases. The number of patients is given in brackets. The number of detected variants is given in square brackets.

Genetic testing was performed in an external laboratory- Blueprint Genetics (Espoo, Finland) on genomic DNA extracted from whole blood. Next-generation sequencing (NGS) multigene panel (MGP) designed for monogenic epilepsies was applied in 123 index cases. Over the 5 years of recruitment the panel has been updated to include novel genes associated with epilepsy, thus the number of analyzed genes varied from 84 to 511 genes. In details, 84 genes were tested in four patients, 128 genes in five patients, 203 genes in four patients, 232 genes in seven patients, 320 genes in 19 patients, 416 genes in 30 patients, and 511 genes in 54 patients. In 16 patients with negative MGP, the analysis was expanded to exome. In the remaining four cases exome sequencing (ES) was performed without prior epilepsy MGP (Figure 1). The average time from sample collection to receiving genetic report was 29 days for MGPs (range 11–51 days) and 51 days for ES (range 29–87 days). The vast majority of epilepsy panels ordered in 2022 and 2023 were completed within 28 days.

All tests included sequence and copy number variation analysis. Detected CNVs were confirmed by quantitative polymerase chain reaction or CMA. Variants were classified according to the guidelines and interpretation criteria established by the ACMG (Richards et al., 2015). Detailed information on sequencing, interpreting and confirmation methods is available online at https://blueprintgenetics.com.

## 3 Results

### 3.1 Clinical characteristics of the studied group

The average age of the patients at the time of genetic testing was 4.3 years (range two months- 18 years). 59 were females and 68 were males. Family history concominant with monogenic inheritance was positive in seven cases. There was no reported consanquinity. The average age of seizure onset was 1.8 years (range three days- 10 years). Of 127 patients recruited, focal-onset seizures were reported in 45 patients, generalized-onset (tonic-clonic, myoclonic, atonic and/or absence) seizures in 51, epileptic spasms in 22, and both focal- and generalized-onset seizures in nine patients. Atypical febrile seizures and/of febrile status epilepticus occurred in 20 patients. Seizures were pharmacoresistant in 53/127 cases. 88 patients had global psychomotor delay. In addition, isolated speech delay was reported in 19 subjects. Intellectual disability was diagnosed in 50 patients and autism spectrum disorder in 21. Dysmorphic features were reported in 23 individuals. In the majority of patients brain MRI was described as normal (93/127). In the remaining 34, the following abnormalities were reported: thinning or agenesis of corpus callosum (*n* = 5), unspecific ventriculomegaly or/and *enlargement* of the subarachnoid *spaces* (*n* = 7), cerebellar hypoplasia/atrophy (*n* = 3), delayed myelination (*n* = 3), nonspecific white matter hyperintensites (*n* = 4), cerebral atrophy (*n* = 3), post-ischemic lesions (*n* = 5), and lesions indicative for a neuronal migration disorder (*n* = 8).

Basing on the type of seizures, anti-seizure treatment response, neurodevelopmental phenotype and EEG recordings we have distinguished five major phenotypic groups: group 1- developmental and epileptic encephalopathies (mostly pharmacoresistant seizures, frequent epileptiform activity on EEG, severe DD or/and ID) (*n* = 40); group 2- developmental delay/regression with generalized or both generalized-onset and focal-onset seizures (seizures partially or fully pharmacoresponsive, mild or moderate developmental delay/ID) (*n* = 41); group 3- developmental delay/regression with focal-onset seizures (focal-onset seizures partially or fully pharmacoresponsive, mild or moderate developmental delay/ID) (*n* = 28); group 4- drug-resistant focal-onset and/or generalized-onset seizures and normal development (*n* = 7); group 5- drug-responsive neonatal seizures, atypical febrile seizures or/and febrile status epilepticus and normal development (*n* = 11).

Summary of clinical characteristics of the studied group is presented in Table 1.

**TABLE 1:** Summary of clinical characteristics of the studied group.

		Positive rates (%)	
Sex			
—	Male	68	24 (35%)
	Female	59	22 (37%)
Age at testing			
	0–3 months	5	3 (60%)
	3–12 months	22	8 (36%)
	1–3 years	46	19 (41%)
	>3 years	54	16 (30%)
Family history concominant with monogenic inheritance		7	3 (43%)
Age of seizure onset			
—	0–3 months	23	9 (39%)
	3–12 months	42	18 (43%)
	1–3 years	33	9 (27%)
	>3 years	29	10 (34%)
Intellectual disability/developmental delay	Global developmental delay	88	36 (41%)
	Intellectual disability	50	24 (48%)
Abnormal brain magnetic resonance imaging	—	34	11 (32%)
Autism Spectrum Disorder	—	21	8 (38%)
Pharmacoresistant seizures	—	53	19 (36%)
Phenotypic group			
—	1- developmental and epileptic encephalopathies	40	16 (40%)
	2- developmental delay/regression with generalized or both generalized and focal seizures	41	17 (41%)
	3- developmental delay/regression with focal seizures	28	10 (36%)
	4- drug-resistant focal and/or generalized seizures and normal development	7	1 (14%)
	5- drug-responsive neonatal seizures, atypical febrile seizures or/and febrile status epilepticus and normal development	11	2 (18%)

### 3.2 Genetic results

A definite molecular diagnosis was established in 46 out of 127 cases (36%). In addition, a highly probable diagnosis was identified in seven additional patients for whom supporting evidence is pending (Table 2). In terms of 41 patients, the diagnosis was identified by MGP, whereas in the remaining five by ES. ES was not preceded by the epilepsy gene panel in four of these patients, whereas in one case it was expanded from a negative MGP. Overall, 16 patients with a negative or inconclusive MGP (*n* = 16/82) had further exome analysis, which provided diagnosis in only one patient (Figure 1).

**TABLE 2:** Summary of genetic test results of the studied group.

ID Sex	Age (y)	Age at seizure onset	Seizure types	Cognitive and behavioral features	Brain MRI	Phenotype	Testing method	Gene	Variant (allele status; transcript; coding DNA; protein)	Classification	Inheritance, parental status
**1 F**	**8**	4 m	Epileptic spasms, generalized myoclonic	GDD, severe ID	Normal	DEE	MGP	*ALG13*	het NM_001099922.2 c.320A>G, p.(Asn107Ser)	Pathogenic	*XL, de novo*
**2 M**	**8**	2 years	Generalized myoclonic, myoclonic-atonic	GDD, ID	Normal	Developmental delay/regression and generalized seizures	MGP	NA	het del(15)(q11.2q12), chr15:g.22646692_28964445	Pathogenic	NA
**3 F**	**8**	3.5 years	GTCS, myoclonic-atonic, absence	GDD, ID	Cerebellar hypoplasia/atrophy	Developmental delay/regression and generalized seizures	MGP	*TPP1*	hom NM_000391.3 c.622C>T, p.(Arg208*)	Pathogenic	AR
**4 F**	**8**	2 years 10 m	FIAS, atypical absence	GDD, ID, ASD	Normal	Developmental delay/regression with focal and generalized seizures	MGP	*MECP2*	het NM_001110792.1 c.2T>C, p.(Met1?)	Likely pathogenic	XL, *de novo*
**5 M**	**8**	2.5 years	Focal motor, FBTCS	GDD, severe ID, ASD	Normal	Developmental delay/regression and focal seizures	MGP	*SLC6A8*	hem c.428_430delACT, p.(Tyr143del)	Likely pathogenic	XL, maternal
**8 F**	**12**	8 m	Generalized myoclonic, atonic, prolonged GTCS	GDD, ID	Mesial sclerosis	DEE	MGP	*KCNA2*	het NM_004974.3 c.1214C>T, p.(Pro405Leu)	Pathogenic	AD, unknown
**15 M**	**9**	6 m	Epileptic spasms, generalized myoclonic	GDD, ID	Post-ischemic lesions	DEE	MGP	*ALDH7A1*	het NM_001182.4 c.328C>T, p.(Arg110*) het NM_001182.4 c.1279G>C, p.(Glu427Gln)	Pathogenic	AR
**17 M**	**8**	4 years	GTCS	GDD	Normal	Developmental delay/regression and generalized seizures	MGP	*TBL1XR1*	het NM_024665.4 c.734A>G, p.(Tyr245Cys)	Likely pathogenic	AD, unknown
**18 F**	**7**	5 m	FIAS, GTCS	GDD, ID	Normal	Developmental delay/regression with focal and generalized seizures	MGP	*SCN1A*	het NM_001165963.1 c.4476 + 6T>G (consequence unknown)	Likely pathogenic	*AD, de novo*
**21 F**	**18**	7 years	GTCS, absence	Normal	Normal	Developmental delay/regression and generalized seizures	MGP	*TBCE*	hom NM_003193.4 c.100 + 1G>A (splice donor variant)	Likely pathogenic	AR
**22 F**	**11**	4 years	Myoclonic-atonic, reflex tonic seizures	GDD, ID, ASD	Normal	DEE	MGP, ES	*NOVA2*	het NM_002516.3 c.826del, p.(Leu276Cysfs*120)	Likely pathogenic	*AD, de novo*
**28 M**	**8**	4 years	FIAS, FBTCS	GDD, mild ID, ASD	Normal	Developmental delay/regression and focal seizures	MGP	*HECW2*	het NM_020760.4 c.2416G>A, p.(Asp806Asn)	Likely pathogenic	*AD, de novo*
**32 M**	**2**	11 m	GTCS, myoclonic, atonic	GDD	Normal	Developmental delay/regression and generalized seizures	MGP	*PCDH19*	het NM_001184880.2 c.1211C>T, p.(Thr404Ile)	Likely pathogenic	XL, SMo
**33 F**	**2**	5 m	Focal motor, FBTCS, FIAS	GDD	Normal	Developmental delay/regression and focal seizures	MGP	*SCN1A*	het NM_001165963.3 c.3637C>T, p.(Arg1213*)	Pathogenic	AD, unknown
**34 F**	**5**	2 years	Generalized myoclonic, myoclonic-atonic	GDD	Normal	Developmental delay/regression and generalized seizures	MGP	*SLC6A1*	het c.(1527 + 1_1528–1)_(*1_?)del	Likely pathogenic	AD, SMo
**35 F**	**6**	1 year 4 m	Epileptic spasms, absence, atonic	GDD, mild ID	Agenesis/thinning of corpus callosum	Developmental delay/regression and generalized seizures	MGP	*WDR45*	het NM_007075.3 c.675del, p.(Lys226Argfs*62)	Likely pathogenic	AD, unknown
**36 F**	**4**	3 m	Epileptic spasms, generalized myoclonic, GTCS	GDD	Nonspecific white matter hyperintensities	DEE	MGP	*SCN1A, SCN2A, SCN3A,SCN9A*	het del(2)(q24.3), chr2:g.165946000-167168500del	Pathogenic	*NA*
**37 M**	**6**	9 m	FIAS	GDD	Normal	Developmental delay/regression and focal seizures	MGP	*CACNA1A*	het NM_001127221.1 c.322A>G, p.(Ile108Val)	Likely pathogenic	*AD, de novo*
**38 M**	**1.5**	4 m	FIAS	Normal	Normal	Drug-resistant focal seizures without DD	MGP	*SCN1A*	het NM_001165963.3 c.4708_4709del, p.(Thr1570Tyrfs*38)	Likely pathogenic	AD, unknown
**41 M**	**6**	10 m	FIAS	GDD, ID, ASD	Normal	Developmental delay/regression and focal seizures	ES	*STXBP1*	het NM_003165.4 c.169 + 1G>A (splice donor variant)	Pathogenic	*AD, de novo*
**44 M**	**5**	3 m	FIAS, GTCS	GDD, mild ID	Normal	Developmental delay/regression with focal and generalized seizures	MGP	*SCN1A*	het NM_001165963.3 c.2791C>A, p.(Arg931Ser)	Likely pathogenic	AD, unknown
**47 F**	**4**	10 m	Generalized tonic, FIAS	GDD	Post-ischemic lesions	DEE	ES	*GABRG2*	het NM_000816.3 c.947C>A, p.(Thr316Asn)	Likely pathogenic	*AD, de novo*
**53 M**	**3**	1 m	Epileptic spasms	GDD	Enlargement of the subarachnoid spaces	DEE	MGP	*STXBP1*	het NM_003165.3 c.1005_1012del, p.(Gln336Glufs*14)	Likely pathogenic	*AD, De novo*
**56 M**	**1**	9 m	Epileptic spasms	GDD	Normal	DEE	MGP	*ACTL6B*	het NM_016188.5 c.1027G>A, p.(Gly343Arg)	Pathogenic	AD, unknown
**58 M**	**6**	9 m	Myoclonic-atonic	Normal	Normal	Developmental delay/regression and generalized seizures	MGP	*SLC6A1*	het NM_003042.3 c.919G>A, p.(Gly307Arg)	Likely pathogenic	AD, unknown
**60 M**	**11**	6 years	FIAS, FBTCS	GDD, severe ID, ASD	Normal	DEE	MGP	*CHD2*	het NM_001271.3 c.4921C>T, p.(Gln1641*)	Likely pathogenic	AD, unknown
**62 F**	**1**	11 m	Focal motor, FIAS, FBTCS	GDD	Neuronal migration disorder	Developmental delay/regression and focal seizures	MGP	*TSC1*	het NM_000368.5 c.733C>T, p.(Arg245*)	Pathogenic	AD, unknown
**64 F**	**6**	5.5 years	FIAS	GDD, severe ID	Normal	Developmental delay/regression and focal seizures	ES	*MECP2*	het NM_004992.3 c.808C>T, p.(Arg270*)	Pathogenic	AD, unknown
**67 M**	**3**	3 m	Generalized myoclonic, focal motor, FIAS, FBTCS	GDD	Normal	Developmental delay/regression with focal and generalized seizures	MGP	*SCN1A*	het NM_001165963.1 c.2589 + 3A>T (the variant destroys the splice donor site in intron 14, leading to the skipping of exon 14 and resulting in greatly reduced protein levels)	Pathogenic	AD, unknown
**72 F**	**5**	6 m	Focal motor, FIAS	GDD, ID	Normal	DEE	MGP	*KCNT1*	het NM_020822.2 c.1193G>A, p.(Arg398Gln)	Pathogenic	AD, unknown
**75 M**	**7**	2.5 years	Focal motor, FIAS, FBTCS	GDD, mild ID	Normal	Developmental delay/regression and focal seizures	MGP	*GRIN2A*	het NM_000833.5 c.1329-1G>T (splice acceptor variant)	Likely pathogenic	AD, unknown
**83 F**	**7**	4 m	Generalized myoclonic, atonic, absence	GDD	Cerebellar hypoplasia/atrophy	Developmental delay/regression and generalized seizures	ES	*PIGT*	het NM_015937.5 c.494–2A>G (splice_acceptor_variant) het NM_015937.5 c.1582G>A, p.(Val528Met)	Pathogenic	AR
**87 M**	**5**	1 m	Focal motor	GDD	Normal	DEE	MGP	*KCNT1*	het NM_020822.2 c.862G>A, p.(Gly288Ser)	Pathogenic	AD, unknown
**89 F**	**4**	3 m	Epileptic spasms	GDD, ID	Cerebellar hypoplasia/atrophy	DEE	MGP	*SLC35A2*	het NM_001042498.2 c.755G>A, p.(Trp252*)	Likely pathogenic	XL, SMo
**92 M**	**3**	5 m	Generalized absence, tonic, atonic	Normal	Unspecific ventriculomegaly and/or enlargement of the subarachnoid spaces	Developmental delay/regression and generalized seizures	MGP	*SLC2A1*	het NM_006516.3 c.971C>T, p.(Ser324Leu)	Pathogenic	AD, unknown
**93 F**	**10**	4 m	Epileptic spasms, generalized tonic	GDD, ID	Normal	DEE	MGP	*EEF1A2*	het NM_001958.5 c.208G>A, p.(Gly70Ser)	Pathogenic	AD, unknown
**96 M**	**2**	1 year 4 m	GTCS	Speech delay	Normal	Developmental delay/regression and generalized seizures	MGP	*SCN1A*	het NM_001165963.3 c.3824G>T, p.(Gly1275Val)	Likely pathogenic	AD, SMo
**97 M**	**13**	9 years	Focal motor, FIAS	GDD, moderate ID	Normal	Developmental delay/regression and focal seizures	MGP	*SYNGAP1*	het NM_006772.3 c.457del, p.(Thr153Profs*21)	Likely pathogenic	AD, unknown
**100 F**	**2**	9 m	Focal motor, FIAS, FBTCS	GDD	Normal	DEE	MGP	*PCDH19*	het NM_001184880.2 c.863_867dup, p.(Gln290Serfs*17)	Likely pathogenic	XL, unknown
**102 F**	**17**	3 m	Prolonged GTCS	GDD, ID	Normal	DEE	MGP	*KCNA2*	het NM_004974.4 c.1220C>G, p.(Pro407Arg)	Pathogenic	*AD, de novo*
**103 F**	**2**	1 year 3 m	Myoclonic-atonic	GDD	Delayed myelination	Developmental delay/regression and generalized seizures	MGP	*SCN8A*	het NM_014191.4 c.4351G>A, p.(Gly1451Ser)	Pathogenic	AD, unknown
**105 M**	**3**	1.5 years	GTCS, atonic	GDD, ASD	Normal	Developmental delay/regression and generalized seizures	MGP	*TPP1*	het NM_000391.4 c.622C>T, p.(Arg208*) het NM_000391.4 c.833A>C, p.(Gln278Pro)	Pathogenic VUS	AR
**110 F**	**0.8**	1 m	Focal motor, FIAS	Normal	Normal	Self-limiting neonatal seizures	MGP	*KCNQ3*	het NM_004519.4 c.1090C>T, p.(Arg364Cys)	Pathogenic	AD, paternal
**115 M**	**16**	5 years	FIAS GTCS	GDD, severe ID	Normal	DEE	MGP	*SCN1A*	het NM_001165963.3 c.1738C>T, p.(Arg580*)	Pathogenic	AD, unknown
**119 F**	**6**	4 years 9 m	FIAS, FBTCS	GDD, moderate ID, ASD	Normal	Developmental delay/regression and focal seizures	MGP	*MECP2*	het NM_004992.3 c.1157_1197del, p.(Leu386Hisfs*5)	Pathogenic	XL, unknown
**124 M**	**0.41**	2 m	Focal motor, FIAS	Normal	Normal	Drug-responsive neonatal seizures	MGP	*DEPDC5*	het NM_001242896.3 c.232del, p.(Arg78Glyfs*2)	Pathogenic	AD, unknown
Patients with highly probable diagnosis											
**10 M**	**9**	6 m	Focal motor, FIAS, FBTCS	GDD, ID, ASD	Normal	Developmental delay/regression and focal seizures	MGP	*SZT2*	het NM_015284.3 c.1444C>T, p.(Arg482Cys) het NM_015284.3 c.4369C>T, p.(Arg1457Trp)	VUS	AR
**42 F**	**4**	10 m	Focal motor, FIAS, FBTCS	GDD	Normal	Developmental delay/regression and focal seizures	MGP	*ATP1A2*	het NM_000702.4 c.994G>C, p.(Glu332Gln)	VUS	AD, unknown
**52 M**	**8**	5 m	Focal motor, FIAS	GDD, severe ID	Thinning or agenesis of corpus callosum unspecific ventriculomegaly or/and enlargement of the subarachnoid spaces; delayed myelination	DEE	MGP	*WWOX*	het NM_016373.3 c.946G>C, p.(Val316Leu) het c.(409 + 1_410–1)_(516 + 1_517–1)del	VUS likely pathogenic	AR
**91 M**	**2**	1 m	Epileptic spasms	Normal	Normal	Neonatal seizures with normal development	MGP	*KCNQ3*	het NM_004519.3 c.1520C>G, p.(Pro507Arg)	VUS	*AD, de novo*
**108 M**	**3**	2 years 9 m	GTCS	Normal	Normal	Atypical febrile seizures without DD	MGP	*SCN1A*	het NM_001165963.3 c.2508C>A, p.(Asp836Glu)	VUS	AD, paternal
**123 F**	**5**	3 m	Focal motor, FBTCS	GDD, moderate ID	Neuronal migration disorder	Developmental delay/regression and focal seizures	MGP	*TSC1*	het NM_000368.5 c.278T>C, p.(Leu93Pro)	VUS	AD, unknown
**126 M**	**0.58**	6 m	Epileptic spasms	GDD	Cerebral atrophy	DEE	MGP	*MT-TL1*	NC_012920.1 m.3243A>G heteroplasmic (level 77%)	Pathogenic	Mitochondrial

AD, autosomal dominant; AR, autosomal recessive; ASD, autism spectrum disorder; DD, developmental delay; DEE, developmental and epileptic encephalopathy; ES, exome sequencing; F, female; FBTCS, focal to bilateral tonic-clonic seizure; FIAS, focal impaired awareness seizure; GDD, global developmental delay; GTCS, generalized tonic-clonic seizure; hem, hemizygous; het, heterozygous; hom, homozygous; ID, intellectual disability; M, male; m, months; MGP, multigene panel; MRI, magnetic resonance imaging; N/A, not applicable; SMo, somatic mosaicism; VUS, variant of unknown significance; XL, X-linked; y, years.

From the 46 individuals with positive results, pathogenic variants were detected in 24 and likely pathogenic in 22. 34 variants have been previously described in the literature and/or disease associated databases, whereas 15 were novel. The variants were predominantly sequence alterations (*n* = 43/46): missense (*n* = 19), nonsense (*n* = 9), frameshift (*n* = 8), splice site (*n* = 6), and one nonframeshift deletion. Pathogenic CNVs were detected in three patients. Mosaicism for the variant was found in four cases. Alterations in six genes have been identified in more than one patient: *SCN1A* (*n* = 7), *MECP2* (*n* = 3), *KCNT1* (*n* = 2), *KCNA2* (*n* = 2), *PCDH19* (*n* = 2), *SLC6A1* (*n* = 2), *STXBP1* (*n* = 2), and *TPP1* (*n* = 2), accounting for 48% of positive cases. In addition, mutations in the following genes were found in single subjects: *ACTL6B, ALDH7A1, ALG13, CACNA1A, CHD2*, *DEPDC5, EEF1A2, GABRG2, GRIN2A, HECW2, KCNQ3, NOVA2*, *PIGT*, *SCN8A, SLC2A1, SLC35A2, SLC6A8, SYNGAP1, TBCE, TBL1XR1, TSC1,* and *WDR45.* Three pathogenic CNVs were detected: del15q11.2-q12 (Angelman syndrome), del(2)(q24.3) encompassing four epilepsy-associated genes (*SCN1A, SCN2A, SCN3A, SCN9A*) and a mosaic deletion c.(1527 + 1_1528–1)_(*1_?)del in *SLC6A1*, encompassing exons 15–16. Autosomal dominant was the main type of inheritance (*n* = 32), followed by X-linked (*n* = 7), and autosomal recessive inheritance (*n* = 5). Summary of genetic test results are given in Table 2.

### 3.3 Positive rates by epilepsy phenotype and age of seizure onset

The highest rate of positive diagnoses was obtained in children with DD/ID and generalized or both generalized- and focal-onset seizures (17/41, 41%) and in children with DEEs (16/40, 40%). In patients with focal-onset seizures and DD/ID a monogenic etiology was identified in 36% (10/28). The lowest diagnostic yields were found in patients with epilepsy and normal development - 18% in the group 5 (2/11) and 14% in the group 4 (1/7).

Regarding the age of seizure onset, the highest rate of positive results was found in patients with seizure onset from three to 12 months (18/42, 43%), followed by birth to 3 months of age (9/23, 39%). In children with first epileptic seizure between one and 3 years of age, DR was 27% (9/33), and over three years- 34% (10/29) (Table 1).

### 3.4 Unexpected findings

Among 46 patients with positive genetic results, 16 were diagnosed with developmental and epileptic encephalopathies. Not surprisingly, *SCN1A* variants were most commonly identified, followed by *KCNT1, KCNA2, PCDH19* and *STXBP1*-related encephalopaties. Various neurodevelopmental disorders with partially or fully pharmaco-responsive epilepsy were also commonly identified (Table 2). Importantly, atypical clinical presentations were found in several cases. In subject#22 clinically diagnosed with DEE exome sequencing performed after a negative MGP detected a *de novo* c.826del, p.(Leu276Cysfs*120) variant in *NOVA2* gene, recently associated with a neurodevelopmental disorder with or without seizures but so far not with DEE. Subject#22 described elsewhere presented severe intellectual disability with lack of speech, global hypotonia and pharmacoresistant sound-induced and unprovoked tonic seizures (Krygier et al., 2022). In subject#41 with moderate intellectual disability and well controlled seizures MGP identified a *de novo* splice donor variant in *STXBP1* gene, classically associated with early infantile epileptic encephalopathy with severe to profound ID (Stamberger et al., 2016). In a 3-year old boy (#108) with normal psychomotor development and several incidents of febrile seizures starting at the age of 19 months we detected a novel heterozygous c.2508C>A, p.(Aps836Glu) variant in *SCN1A* gene, classified as variant of unknown significance. Interestingly, his twin brother who experienced three incidents of febrile seizures died suddenly during sleep at the age of 2.5 years. The same variant was identified in their father with normal intellectual development and epilepsy with generalized tonic-clonic seizures from 20 years of age, which was effectively treated with valproate (VPA) for several years and the treatment was withdrawn with no seizure recurrence. Brother of the father had profound ID with lack of speech and epilepsy from the second year of life, and he died unexpectedly at the age of 21 years during sleep. The father’s sister with severe ID, in whom we also detected the c.2508C>A, p.(Aps836Glu) *SCN1A* variant has been suffering from epilepsy from the first year of life. Her neurological examination at the age of 18 was notable for parkinsonian features (bradykinesia, resting tremor) and crouch gait pattern consistent with Dravet syndrome. Epilepsy with generalized tonic-clonic seizures has been treated from fourth year of life, initially with carbamazepine (CBZ) and later with VPA and topiramate for 7 years. From the last 2 years the patient has been receiving VPA and CBZ and generalized tonic-clonic seizures occurred approximately once per one to 2 weeks. After the diagnosis of Dravet syndrome CBZ was withdrawn and clobazam was implemented, which lead to prompt increase in seizure frequency (once per 2 days). Therefore, therapy with VPA and CBZ was resumed, leading to significant improvement in daily activity and seizure control.

Lastly, in the case of two patients with focal epilepsy and developmental delay (#62, #123) genetic testing allowed a diagnosis of tuberous sclerosis complex (TSC), despite lack of previous clinical and neuroimaging suspicion of TSC. Patient #62is a 17-month-old girl with focal-onset seizures not controlled on two anti-epileptic drugs (valproate, levetiracetam), and a few unspecific focal lesions in the lateral ventricles and white matter with *high signal* on *T1*-*weighted* brain MRI images. Physical examination revealed no skin lesions and prenatal heart ultrasound and postnatal abdominal ultrasound examinations were normal. MGP identified a pathogenic.733C>T, p.(Arg245*) variant in *TSC1* gene. Parental studies were unavailable due to oocyte donation. Currently the seizures are well controlled on monotheraphy with vigabatrin. Patient #123 is a 5-year-girl with drug-resistant focal seizures from third month of age, global DD, ataxia, and MRI findings, repeatedly described as post-ischemic lesions. MGP identified a novel c.278T>C, p.(Leu93Pro) variant in *TSC1* gene, classified as VUS. Further clinical evaluation revealed two skin hypomelanotic macules and one café au lait spot, no heart alterations and several bilateral simple kidney cysts. MRI images were consulted with specialists experienced with TSC, who confirmed the presence of migration and hamartomatous brain lesions in the patient.

### 3.5 Treatment implications

53/127 patients developed pharmacoresistant epilepsy, 19 of whom (36%) had a single-gene etiology identified. Overall, molecular genetics diagnosis has led to change in anti-seizure management in 15 out of 46 cases (33%). These were mainly patients with *SCN1A*-related epilepsy (7/15), who have benefited from the introduction of first-line and add-on therapy for Dravet syndrome and/or halting carbamazepine for focal seizures. Specific treatment strategies were also implemented for patients with Glut1 deficiency syndrome (ketogenic diet and withdrawal of ASM), pyridoxine-dependent epilepsy (large daily supplements of pyridoxine), creatine transporter deficiency (supplementation with creatine), and neuronal ceroid lipofuscinosis (enzyme replacement therapy). In addition, one patient (#62) with the pathogenic *TSC1* variant became seizure-free after switching to monotherapy with vigabatrin and one patient with *GRIN2A* splice-site variant started supplementation with L-serine (#75).

## 4 Discussion

This is the first and the largest study assessing the genetic landscape of epilepsy in Poland. The results show extensive genetic heterogeneity with only two genes detected in more than two individuals (*SCN1A* and *MECP2*). The overall diagnostic yield of NGS in our cohort (36%) is high in comparison to other studies, which can be attributed to the inclusion criteria, which selected children with suspected monogenic etiology and a large number of genes included in the MGP (Sánchez Fernández et al., 2019; Stefanski et al., 2021; Sheidley et al., 2022). According to the recent systematic review an average diagnostic yield of MGP in epilepsies is 19% (95% confidence interval [CI] = 16%–24%) (Sheidley et al., 2022). However, the majority of the studies have assessed the yields of smaller MTPs, which correlates with lower positive rates. Interestingly, in this review the yields of large MGPs (>500 genes) were higher than the yields of ES, which raised the question whether large gene panels should be used as a first-tier testing. Of note, our study showed only a small diagnostic superiority of ES over panel testing. This indicates that large MGPs have high diagnostic sensitivity and should be considered a first-line genetic test in clinically defined epilepsy syndromes, in which monogenic etiology is highly probable. Although the recent practice guideline of the National Society of Genetic Counselors recommends ES/GS over MGP as the first-tier test, it should be noted that the access to ES/GS in many regions is still limited and a long turnaround time may be not acceptable (Smith et al., 2023). MGPs enable rapid and cost-effective diagnosis in clinical setting, which is especially important in the view of a growing number of potentially treatable epilepsies (Tumiene et al., 2022). At this point, the value of metabolic testing in epilepsies cannot be omitted. CMA should be considered a second-line genetic test, unless the phenotype is highly suggestive for chromosomal aberration.

At the molecular level our results show high diversity in disease-causing alterations and a high rate of previously unreported variants (15/39). In line with the literature, the most common mutations were single-nucleotide variations (SNVs) and small insertions or deletions (indels) localized in the coding or splice-site regions (Hamdan et al., 2017). CNVs were found in only 3/46 cases which is relatively low in comparison to other studies, however it should be noted that patients with DD and epilepsy are frequently initially referred for CMA testing before NGS. Importantly, 4/46 cases (8.7%) were mosaic for the variant. Somatic mosaic mutations arise postzygotically and are present only in a subset of the cells of an affected individual. Somatic mosaicism is an obligatory disease mechanism in several overgrowth syndromes, such as *Proteus* syndrome, Sturge-Weber or hemimegalencephaly, but is also frequently identified in other neurodevelopmental disorders, like neuronal migration disorders, neurocutaneous syndromes, intellectual disabilities, and ASD (D’Gama and Walsh, 2018). In the epilepsies, for several years genetic testing has focused primarily on the detection of inherited and *de novo* germline variants which represent the majority of disease-causing variants. However, in the last decade the advent of deep sequencing techniques generating high read depths and advances in bioinformatics analyses has shown the importance of somatic mosaicism in both lesional and non-lesional epilepsies. Non-brain-limited somatic mosaicism arising during early development can be detected in blood on condition that the variant allele frequency is high enough to be detected by a testing method (D’Gama and Poduri, 2023). It is important to emphasize that the level of mosaicism between different tissues may vary and mosaic pathogenic variants may lead to a spectrum of clinical phenotypes ranging from mild to severe, depending on the tissues affected. Thus, there is a necessity to test other clinically accessible tissues, such as fibroblasts or buccal swab speciments to inform prognosis and management. Unfortunately, such samples were not available in our study. Currently is difficult to assess the frequency of non-brain-limited somatic mosaicism in epilepsy as the majority of reports in the literature represent single cases. Stosser *et* al. analyzed almost 900 patients with pathogenic variants in nine genes associated with epilepsy and found that 3.5% were actually mosaic for the variant, most commonly in *CDKL5, PCDH19, SCN2A,* and *SCN1A* genes (Stosser et al., 2018). Chen *et* al. identified somatic mosaic variants in 20 out of 264 probands with monogenic epilepsy using deep-amplicon variant sequencing (Chen et al., 2023). Of note, we identified a likely pathogenic mosaic variant in PCDH19 *gene in a boy presenting with tonic seizures at the age of 11 months (#32) supporting* cellular interference as the pathogenic mechanism in symptomatic males with *PCDH19*-related encephalopathy (Depienne et al., 2009). We also identified a novel large mosaic *SLC6A1*deletion in a girl with myoclonic-astatic epilepsy (#34). So far, somatic mosaicism for *SLC6A1* variant has only been described in asymptomatic mothers of affected individuals (Halvorsen et al., 2016; Poliquin et al., 2021). The frequency of somatic mosaicism detected in our cohort (8.7% of positive cases) highlights the importance of deep sequencing in clinical genetic testing for the epilepsies.

The results of our study show that the major factor that impacts the rate of positive diagnoses in epilepsy is the presence of developmental delay and/or intellectual disability. Surprisingly, a comparable DR was achieved in patients with the most severe epileptic phenotype (DEE) and in subjects with partially or fully pharmacoresponsive generalized seizures and milder neurodevelopmental deficits. Noteworthy, a high DR was also identified in subjects with solely focal-onset seizures (34%). This can be partially attributed to the high extent of *SCN1A*-related epilepsy not manifesting as DEE in our cohort. From seven patients with *SCN1A* variants, four were classified to the group 2, one to the group 1, and two to the group 3, presenting solely focal seizures (#33,#38). This is consistent with a recent study, showing that focal epilepsies are a frequent feature of *SCN1A*gain of function variants beyond Dravet syndrome and generalized epilepsy with febrile seizures plus. In such case, sodium channel blockers may be beneficial in controlling seizures by inhibiting excessive channel function (Matricardi et al., 2023). This mechanism may be responsible for the worsening of seizure control after CBZ withdrawal in the relative of subject#108, though functional effect of the novel c.2508C>A *SCN1A* variant remains unknown. In terms of channelopathies, determining the functional consequence of a variant (loss-of-function *versus* gain-of-function) is crucial to guide therapeutic decisions and to pave the way towards precision medicine.

## 5 Study limitations

This study should be interpreted in light of several limitations. First, it included a relatively small and selected group of children with suspected monogenic epilepsy. Therefore, diagnostic yields may be overestimated in comparison to studies with broader inclusion criteria. The sample size being small, caution should be exercised when interpreting the results in relation to broader populations. Second, the extend of molecular genetic testing varied between individuals, which may significantly impact the rate of positive diagnoses. The diversity of commercially available epilepsy gene panels poses a risk for some epilepsy-associated genes being not included in a particular panel. On the other hand, testing a broad number of genes is associated with longer duration of analysis and higher likelihood of identifying VUS in genes unrelated to a patient’s phenotype (Chambers et al., 2016). Last, phenotypic subgroups were distinguished based on predominant clinical features, which may be influenced by vide heterogeneity and overlap in clinical symptoms, such as different seizure types and EEG abnormalities evolving over time.

## 6 Conclusion

Our results demonstrate the high diagnostic and therapeutic utility of next-generation sequencing testing in patients with childhood-onset epilepsies. Although monogenic etiologies are most commonly identified in DEEs and generalized epilepsies, high rates of positive diagnoses are also detected in children with focal seizures and neurodevelopmental concerns. Importantly, copy number variations and somatic mosaic variants are disease-causing factors in a substantial proportion of cases. Thus, comprehensive genetic testing, including deep sequencing and copy number variation analysis is recommended in all unexplained cases. Especially since misinterpretation of clinical and neuroimaging data may significantly influence patients diagnosis and management. Pleiotropy is a common phenomenon contributing to the extensive phenotypic variability of single-gene epilepsies. Moreover, diverse functional outcomes and variable expressivity of gene alterations even within the same family reflects the growing complexity of genotype-phenotype associations.

## Data Availability

The original contributions presented in the study are publicly available. This data can be found here: https://www.ncbi.nlm.nih.gov/clinvar/ with the following accessions SUB13951534, SUB13954780, SUB13951519, SUB13951496, SUB13951459, SUB13954818, SUB13951510, SUB13954751, SUB13951540, SUB13954774, SUB13951527, SUB13954737, SUB13954828, SUB13954909, SUB13954881, SUB13954938, SUB13954927, SUB13954899, SUB13954845, SUB13954757, SUB13958441, SUB13954966, SUB13958464.
